# Immune and Neural Response to Acute Social Stress in Adolescent Humans and Rodents

**DOI:** 10.21203/rs.3.rs-3845793/v1

**Published:** 2024-02-05

**Authors:** Vilma Gabbay, Benjamin Ely, Julia Vileisis, Zorica Petrovic, Ana Cicvaric, Gregory Asnis, Seunghee Kim-Schulze, Jelena Radulovic

**Affiliations:** University of Miami; Albert Einstein College of Medicine; Albert Einstein Medical College; Albert Einstein College of Medicine; Icahn School of Medicine at Mount Sinai

## Abstract

Studies in adults have linked stress-related activation of the immune system to the manifestation of psychiatric conditions. Using a translational design, this study aimed to examine the impact of social stress on immune activity in adolescents and on neuronal activity in a preclinical mouse model. Participants were 31 adolescents (ages 12–19), including 25 with mood and anxiety symptoms. Whole-blood samples were collected before and after the Trier Social Stress Test (TSST), a stress-inducing public speaking task, then cultured for 6 hours in the presence and absence of the inflammatory endotoxin lipopolysaccharide (LPS). Effects of TSST and LPS on 41 immune biomarkers were examined using repeated-measures analysis of variance. Separately, juvenile (8-week-old) male mice were non-stressed or exposed to reminder social defeat then intraperitoneally injected with saline or LPS (n = 6/group). Brains were perfused and collected for immunohistochemistry and confocal microscopy at 0, 1, 6, and 24 hours post-injection. Activity was determined by the density of cFos-positive neurons in the paraventricular hypothalamus, paraventricular thalamus, and basolateral amygdala, regions known to show sustained activation to immunological challenge. Analyses in the adolescent study indicated a strong effect of LPS but no effects of TSST or TSST×LPS interaction on immune biomarkers. Similarly, reminder social defeat did not induce sustained neuronal activity changes comparable to LPS immunological challenge in juvenile mice. Our convergent findings across species suggest that the acute immune response to stress documented in adults is not present in youth. Thus, aging and chronicity effects may play an important role in the inflammatory response to acute psychosocial stress.

## INTRODUCTION

Immune system dysregulation has been implicated in the development of psychiatric conditions, including depression and anxiety. One postulated mechanism is that psychological stress induces a systemic inflammatory reaction that affects brain function. Preclinical data have demonstrated that exposure to an immunological challenge, such as the endotoxin lipopolysaccharide (LPS), results in “sickness behavior” characterized by decreased social exploration, sleep disorders, and inhibition of sexual behavior [1–6]. Similarly, clinical studies by other groups and our team have reported increased levels of circulating immune mediators in both adult and pediatric patients with depression and other disorders [7–16].

To understand better immune system dysregulation in response to acute psychological stress, several studies in adults and youth have used the Trier Social Stress Test (TSST) [17], where participants complete public speaking tasks in a laboratory setting. The TSST is known to increase salivary cortisol, heart rate, heart rate variability, systolic blood pressure, and negative affect in youth, all markers for increased stress [18]. The TSST has also been shown to induce an immune response in both healthy and clinical adult populations [14, 19–21]. A meta-analysis of 30 studies found increased circulating plasma cytokines following acute social stressors [22], and many groups have reported increased interleukin (IL)-6 [23–28] [25–30], tumor necrosis factor (TNF)-α [28, 29], IL-1RA [21], IL-1β [29], and IL-10 [29] in response to the TSST. However, there have also been studies that failed to show reliable changes in IL-6 [19, 30–33] and TNF-α [32, 34] in response to the TSST. Moreover, no studies to date have evaluated the effects of the TSST on cytokine production in adolescence, when many psychiatric conditions first emerge. Since the TSST is widely used to investigate immune system activation in psychiatric cohorts, it is important to validate its effect on the immune system in youth. Given the divergent effects of acute vs. chronic psychological stress on immune function, it is also critical to examine these systems early in the course of psychiatric conditions, prior to chronicity effects.

Building upon the above observations, we sought to identify the effects of acute psychological stress on immune function in adolescents with mood and anxiety symptoms using the TSST. As these symptoms are common across psychiatric disorders, we adopted the NIMH Research Domain Criteria (RDoC) approach of studying adolescents with diverse clinical symptoms, including comorbid and subthreshold diagnoses. Healthy controls were also included to provide a full range of symptom severity and because studies in adults have documented similar immunological effects of the TSST in participants with or without psychiatric conditions. All participants were psychotropic-medication-free. We hypothesized that in adolescents, like in adults, the TSST would result in acute immune system activation. To test this, we assessed a detailed panel of 41 cytokines, chemokines, and growth factors using multiplex assays to capture the complex, multifactorial nature of the immune system, as in our prior studies [7, 8]. Whole-blood samples were collected immediately before and after administering the TSST. Since LPS reliably induces an acute proinflammatory cytokine response through stimulation of toll-like receptor 4 [35], samples were cultured both with and without LPS before being assayed. This 2×2 design enabled us to characterize the immune response to acute stress, verify the expected immune response to LPS as a positive control, and evaluate the relative magnitudes of these responses *in vitro*. Additionally, to examine whether the effects of acute psychosocial stress mimic the effects of acute systemic immune activation on brain activity, we conducted a complementary preclinical experiment in juvenile mice. To this end, we measured the timecourse of cFos activation in the hypothalamus, thalamus, and amygdala after an acute psychosocial stressor (reminder social defeat paradigm) or an acute immune challenge (intraperitoneal LPS injection). This design allowed us to directly compare the acute effects of social vs. immune stress on brain regions known to show sustained response to immune activation in rodents.

## PATIENTS AND METHODS

### Human Study

#### Participants

Adolescents, ages 12–19, were recruited in the greater New York City area. Participants under age 18 provided written assent, and a parent or guardian gave written informed consent; participants 18 years and older provided written informed consent. The study was approved by the Institutional Review Board of Icahn School of Medicine at Mount Sinai.

#### Inclusion and Exclusion Criteria

Inclusion criteria for adolescents with psychiatric symptoms: presence of one or more mood or anxiety symptoms based on diagnostic evaluation. Symptoms were clinically significant but were not required to meet the severity threshold for a full diagnosis.

Inclusion criteria for healthy control adolescents: no past or current psychiatric conditions or clinically significant symptoms.

Exclusion criteria for all participants: 1) any physical or neurological conditions; 2) estimated IQ<80; 3) a positive drug toxicology test; 4) a positive pregnancy test; 5) current psychosis, pervasive developmental disorder, or substance abuse; 6) psychotropic medication use in last 1–3 months at baseline visit, depending on drug half-life; 7) any recent inflammatory illnesses, including the common cold; and 8) any recent anti-inflammatory medication use, including over-the-counter remedies.

#### Clinical Assessments

All participants received diagnostic evaluations using the Schedule for Affective Disorders and Schizophrenia for School-Age Children–Present and Lifetime Version (KSADS-PL) [36], administered by a trained child and adolescent psychiatrist or a clinical psychologist. Additional assessments included the Children’s Depression Rating Scale-Revised (CDRS-R) [37] to measure the presence and severity of depression symptoms and the Kaufman Brief Intelligence Test (K-BIT) [38] to estimate overall IQ. Clinical symptom severity was quantified using the self-rated Beck Depression Inventory (BDI) [39] and Multidimensional Anxiety Scale for Children (MASC) [40].

#### Trier Social Stress Test Procedures

Participants arrived in the early morning after fasting ≥12 hours. A small cannula was inserted into the vein to allow for two blood draws. The first was conducted before the TSST and the second was conducted within 5–10 minutes after TSST completion. Participants completed a slightly modified version of the TSST, designed for children and adolescents [41], composed of two sections: a brief preparation period followed by a mock interview in which the participant told a story and performed mental arithmetic before a panel of three adult judges. The youth TSST protocol thus maintains important elements of a stress-inducing task, including a threat to the social self, uncontrollability, and unpredictability. This protocol has been shown to raise blood pressure, heart rate, and salivary cortisol in children and adolescents, even when controlling for the novelty of visiting a laboratory [42], and has been shown to reliably induce stress in adolescent populations in clinical settings [43]. Following the TSST, participants rated how anxious they felt during the task from 1 (“Not at all”) to 6 (“Extremely”) using a visual analog scale (VAS).

#### Immune Biomolecule Assessment

Procedures follow those described in our previous immunological studies [7,8]. Whole-blood samples were cultured for six hours under two conditions: using standard culture medium alone and using standard culture medium plus LPS (0.1μg/ml). Supernatants were then harvested and analyzed for immune biomarkers using a Luminex-200 system and the xMap Platform (Luminex Corporation, Austin TX) following manufacturer recommendations. Levels were determined in duplicate 25μL volumes of supernatant using multiplex panels (Multiplex High Sensitivity 41-plex Human Cytokine/Chemokine Panel, Millipore Corp.) and reported as median fluorescence index (MFI) values; analyses used the mean of the two duplicate MFI measurements per analyte. Lower and upper detection limits for assays were 3.0pg/mL and 15ng/mL, respectively. Multiplex assays targeted 41 unique immune biomolecules (see Table 2), including the cytokines IL-1β, IL-2, IL-4, IL-6, IL-8, IL-10, IL-12, interferon (IFN)-γ, and TNF-α.

#### Statistical Analyses

Immune activation was evaluated using Matlab 2017a. Lilliefors goodness-of-fit tests indicated that levels of most biomarker were not normally distributed in our sample. Data were therefore normalized using the Box-Cox method to allow the use of parametric analyses. The effects of TSST, LPS, and TSST×LPS interaction were then tested using repeated-measures analysis of variance (rANOVA) in a within-subject 2×2 design, with diagnostic status (clinical or healthy control participant), age, sex, and body mass index (BMI) included as covariates. To further evaluate potential associations between immune biomarkers and TSST-induced stress, rANOVA analyses were repeated including participants’ self-reported anxiety during the TSST (1–6 VAS scores) as an additional covariate. Results were controlled for multiple comparisons using Bonferroni correction (two-tailed *p*_*Bonferroni*_≤0.05/41≈1.2×10^−3^). Complete subject-level data used for rANOVA, including covariates and analyte levels at each timepoint/condition, are provided in **Supplementary Tables S1-S4**. *Post hoc* sensitivity analyses conducted in G*Power v3.1.3 indicated 96% power to detect moderate effects (Cohen’s *f*=0.25) and 80% power to detect effects as small as *f*=0.20 in our sample.

### Animal Study

#### Mice

All experimental protocols were approved by the Institutional Animal Care and Use Committee of Albert Einstein College of Medicine. Seven-week-old male experimental mice (C57BL/6N) were purchased from Envigo. CD-1 male retired breeders over 16 weeks of age were purchased from Charles River. After arriving to our colony, all animals were single housed with *ad libitum* access to food and water on a 12hr light/dark cycle (7AM-7PM). The experiment was performed after one week of acclimatization in our satellite facility. CD-1 mice were kept in separate room from the experimental mice to avoid habituation to their odors. Prior to the experiment, CD-1 mice were screened for aggressive behavior to identify viable resident aggressors.

The social stress paradigm was performed when experimental mice were 60 days (eight weeks) old, which corresponds to an approximate human age of 16 years (see Ch. 20 in [44]) and may be considered late adolescence or early adulthood, depending on the source. This age was chosen based on previous findings that acute social stress paradigms reliably induce behavioral changes in mice aged 50–61 days but not in younger mice aged 29–40 days [45,46]. Similarly, LPS exposure increases neural cFos expression and serum corticosterone levels in young adult mice aged 70 days but not in peripubertal mice aged 42 days [47]. Experimental mice were limited to males to ensure the validity of the social defeat paradigm, which does not readily generalize to females [48].

Mice were randomly assigned to one of three main experimental groups, detailed below: non-stressed (NS) mice receiving injections of saline (NS-SAL) or LPS (NS-LPS) and mice exposed to the reminder social defeat (SD) paradigm receiving saline injections (SD-SAL). Timecourse data were collected at 0hr, 1hr, 6hr, and 24hr post-injection. The 6hr timepoint also included a fourth experimental group of mice exposed to the reminder SD paradigm and given LPS injections (SD-LPS). Six mice were assigned to each group at each timepoint (*N*=78 total).

**Figure 1:**
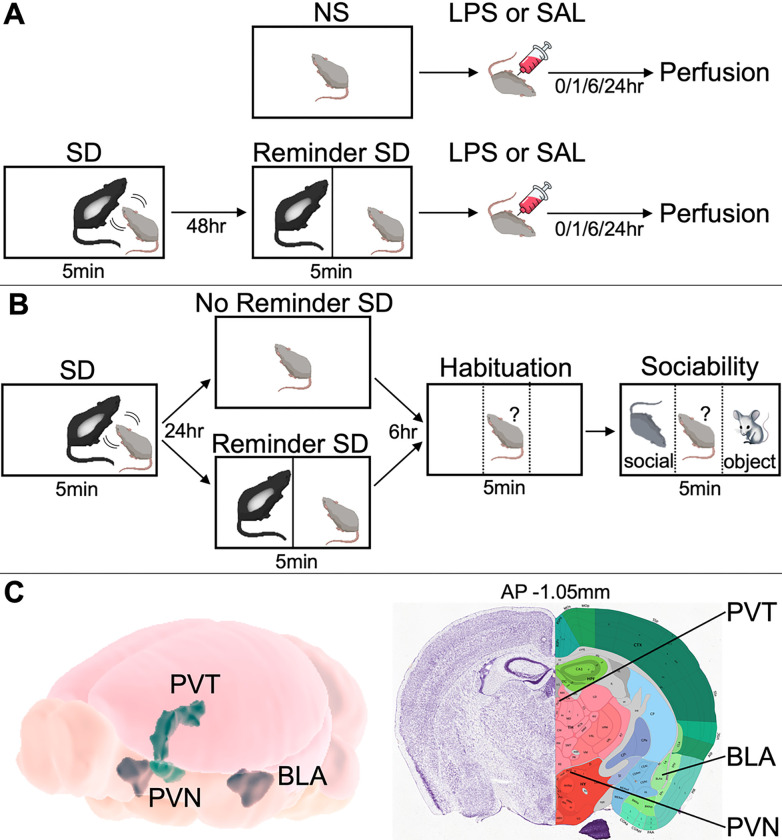
(**A**) Schematic of mouse behavioral paradigm. Top: non-stressed (NS) mice i.p. injected with saline (NS-SAL) or LPS (NS-LPS). Bottom: stressed mice exposed to 5min of social defeat (SD), followed 48hr later by 5min of reminder SD, then i.p. injected with saline (SD-SAL) or LPS (SD-LPS, 6hr timepoint only). Perfusion and tissue extraction performed 0hr, 1hr, 6hr, or 24hr after injection. (**B**) Behavioral paradigm consisting of SD on day 1, reminder SD the next day in half of the mice, and assays to measure habituation (side preference) and sociability (social vs. object preference) 6hr later in all mice. Location of conspecific (social) and toy (object) mice was counterbalanced. (**C**) Target regions for cFos quantification. Left: 3D depiction of paraventricular hypothalamic nucleus (PVN), paraventricular thalamic nucleus (PVT), and basolateral amygdala (BLA). Right: Nissl-stained coronal mouse brain section and superimposed atlas depicting PVN, PVT, and BLA. Image credit: Allen Brain Atlas.

An overview of experimental procedures in the animal study is provided in Figure 1A.

#### Reminder Social Defeat Paradigm

The social stress paradigm was an acute version of the reminder SD protocol [49]. Each experimental C57BL/6N mouse in the SD branch was introduced to the cage of a resident CD-1 aggressor for five minutes, during which the intruders were attacked and exhibited avoidance, submissive, and defensive behaviors (SD exposure). After 48hr, each experimental mouse was returned to the same resident aggressor’s cage for 5min but placed behind a partition, enabling the experimental mouse to smell and see the dominant CD-1 mouse but preventing physical contact between the animals (“reminder” SD exposure). Mice in the non-stressed (NS) branch were not exposed to resident CD-1 aggressors.

#### Behavioral Validity of Reminder Social Defeat Paradigm

Behavioral validity data for the reminder SD paradigm are included from a separate cohort of 20 male C57BL/6N mice, illustrated in Figure 1B. Mice were introduced to the cage of a resident CD-1 aggressor for 5min (SD exposure). After 24hr, half of mice were returned to the same resident aggressor’s cage behind a partition for 5min (reminder SD exposure) and half received no reminder SD exposure. After another 6hr, behavioral assays were performed on all mice. Habituation was assessed by returning mice to the same resident aggressor’s cage alone for 5min and measuring the fraction of time spent on each side. Sociability was then assessed for 5min by placing a conspecific mouse on one side of the cage and a toy mouse on other side of the cage and measuring the fraction of time spent on each side. Locations of conspecific and toy mice were counterbalanced.

#### Induction of Systemic Inflammation

Following completion of the reminder SD protocol, mice in the LPS branch received a single intraperitoneal (i.p.) injection with LPS (Escherichia coli 005: B5, No. L-2880 Sigma-Aldrich, St. Louis MO) at a dose of 0.83mg/kg dissolved in 200μl of sterile, pyrogen-free physiological saline. LPS dose was selected to induce acute systemic inflammation based on earlier findings [50,51]. Mice in the SAL branch received an equivalent i.p. injection of saline alone as a negative control. Experimental mice were euthanized 0hr, 1hr, 6hr, and 24hr after injection in the NS-SAL, NS-LPS, and SD-SAL groups and 6hr after injection in the SD-LPS group.

#### Immunohistochemistry of cFos

Mice were anesthetized with an i.p. injection 1ml Avertin (480mg/kg of tribromoethanol/isopropyl alcohol) and transcardially perfused with ice-cold 4% paraformaldehyde in phosphate buffer (pH=7.4). Brains were removed and post-fixed for 24hr in the same fixative and then immersed in 20% and 30% sucrose in phosphate buffer for 24hr each. Brains were frozen for 1hr at −80°C in Tissue-Plus^™^ O.C.T. Compound (Thermo Fisher Scientific, Waltham MA) and 50μm sections were cut. For antigen retrieval, sections were incubated in citrate buffer (pH=6.0) for 30min at 70°C, washed 3× in phosphate-buffered saline (PBS), and blocked in PBS containing 0.3% Triton X-100 and 5% normal goat serum. Sections were incubated overnight at 4°C in cFos antibody 1:2000 (Abcam, ab208942). After incubation, sections were washed in PBS containing 0.2% Triton X-100 and 1% normal goat serum, then incubated for 2hr at room temperature in Alexa Fluor^®^ 488 AffiniPure donkey anti-rabbit IgG (H+L) 1:500 and 594 AffiniPure donkey anti-mouse IgG (H+L) 1:400 (Jackson Immuno Research, West Grove PA, 711-545-152 and 715-585-150). Nuclei were counterstained with Hoechst 33342 1:3000 (Thermo Fisher Scientific). Prepared sections were slide mounted and cover-slipped with Vectashield (Vector Laboratories, Burlingame CA). Sections were observed on a Leica fluorescent microscope and Olympus Fluoview confocal microscope using 10× and 60× objectives.

As illustrated in Figure 1C, images were captured at the level of paraventricular hypothalamic nucleus (PVN), paraventricular thalamic nucleus (PVT), and basolateral amygdala (BLA). These regions were selected due to their involvement in stress response [52,53] [54,55] as well as published [54] and in-house data showing that these areas exhibit sustained upregulation of cFos following acute immune challenge. Quantification was performed using ImageJ, as described previously [55]. For cFos, cell counts were performed using a constant threshold (diameter >5μm, intensity >0.47a.u.). All quantification performed blind to experimental group using two sections per mouse, unless prohibited by lesion.

#### Statistical Analyses

Mouse immunohistochemical data were analyzed using Graphpad. All samples were normally distributed, as determined with a one-sample Kolmogorov-Smirnov test. Homogeneity of variance was confirmed with Levene’s test for equality of variance. The results were analyzed using two-way ANOVAs with group and timepoint as factors. For the 6hr timepoint, additional two-way ANOVAs were run with stressor (NS, SD) and injection type (SAL, LPS) as factors. Significant F values were followed by *post hoc* pairwise *t*-tests. All tests were run separately for each region and Bonferroni adjusted for multiple comparisons (two-tailed *p*_*Bonferroni*_≤0.05/3≈0.017). Power considerations for the animal study were informed by our recent work [56–58] [58–60] as well as pilot experiments. Minimum power to detect anticipated effects was set at 80% (1-*β*>0.80; two-tailed *α*≤0.05). Sensitivity analyses performed using G*Power v3.1.3 indicated power to detect moderate-to-large group (*f*=0.37) and timepoint (*f*=0.40) effects in our sample.

## RESULTS

### Adolescent Sample Characteristics

Participant demographic and clinical characteristics are compiled in Table 1. The sample consisted of 31 unmedicated adolescents, including 25 with psychiatric symptoms and 6 healthy controls. All participants were included in analyses. Immunological data from pre-TSST blood samples collected in this cohort were included in two prior studies [7,8]; no data from post-TSST blood samples have been published previously.

### Behavioral and Immune Response to Acute Social Stress and Inflammation in Adolescents

Results from the rANOVA, compiled in Table 2, indicated no effect of TSST on immune biomarker levels at Bonferroni-corrected (*p*≤1.2×10^−3^) or even uncorrected (*p*≤0.05) statistical thresholds. By contrast, LPS induced significant changes in the levels of 22 out of 41 immune biomarkers, consistent with an acute inflammatory response. No significant TSST×LPS interaction was detected (all *p*>0.1). For covariates, rANOVA indicated a significant TSST×sex interaction for a single analyte, MCP-1 (*p*=7.3×10^−4^; *f*=0.39); at a relaxed threshold of *p*<0.01, two additional TSST×LPS×diagnosis interactions were detected for IL-3 (*p*=3.3×10^−3^; f=0.30) and IL-15 (*p*=7.9×10^−3^; *f*=0.25). No effects of age or body mass index (BMI) were observed. Similarly, although the TSST successfully elicited a subjective stress response from participants (4.0±1.5 on six-point VAS; *p*<10^−4^), we found no effect of TSST-induced anxiety scores when included as an additional covariate in secondary analyses.

### Neural Response to Acute Social Stress and Inflammation in Mice

**Figure 2:**
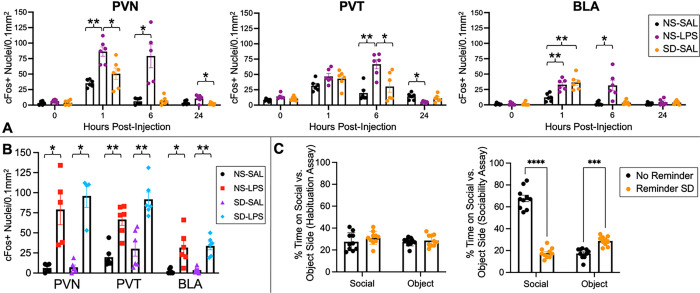
Quantification of cFos responses in the PVN, PVT, and BLA for the (**A**) NS-SAL, NS-LPS, and SD-SAL experimental groups across the 0hr, 1hr, 6hr, and 24hr timepoints, with (**B**) additional data from the SD-LPS experimental group at the 6hr timepoint. (**C**) Behavioral validity experiment results. Mice that received reminder SD vs. SD with no reminder showed no difference in side preference during the habituation assay but significantly increased preference for object interactions and reduced preference for social interactions during the sociability assay. Plots show mean±S.E.M. overlaid with individual datapoints. Significant changes indicated as *p≤0.05, **p≤0.01, ***p≤0.001, ****p≤0.0001

Timecourse data for cFos expression in mice were analyzed using two-way ANOVA to examine the effects of group (NS-SAL, SD-SAL, NS-LPS), timepoint (0hr, 1hr, 6hr, 24hr), and group×timepoint interaction on cFos expression. Separate ANOVA models were run for each region (PVN, PVT, BLA) and corrected for multiple comparisons. Findings indicated that group, timepoint, and interaction effects were highly significant for each region: PVN group (*F*_*2,59*_=37.0; *p_Bonferroni_*<10^−5^), timepoint (*F*_*3,59*_=53.8; *p*_*Bonferroni*_<10^−5^), and interaction (*F*_*6,59*_=10.0; *p*_*Bonferroni*_<10^−5^); PVT group (*F*_*2,59*_=10.2; *p*_*Bonferroni*_=4.7×10^−4^), timepoint (*F*_*3,59*_=39.5; *p*_*Bonferroni*_<10^−5^), and interaction (*F*_*6,59*_=7.43; *p*_*Bonferroni*_=2×10^−5^); and BLA group (*F*_*2,60*_=16.2; *p*_*Bonferroni*_<10^−5^), timepoint (*F*_*3,60*_=38.6; *p*_*Bonferroni*_<10^−5^), and interaction (*F*_*6,60*_=8.56; *p*_*Bonferroni*_<10^−5^). Post hoc tests compared cFos expression pairwise across groups for each timepoint, as shown in Figure 2A. These tests indicated that cFos expression was significantly elevated: at the 1hr timepoint for NS-LPS vs. NS-SAL in the PVN (*p*_*Bonferroni*_=2×10^−3^; *d*=3.7) and BLA (*p*_*Bonferroni*_=4×10^−3^; *d*=2.6), for SD-SAL vs. NS-SAL in the BLA (*p*_*Bonferroni*_=7×10^−3^; *d*=2.6), and for NS-LPS vs. SD-SAL in the PVN (*p*_*Bonferroni*_=0.04; *d*=1.7); at the 6hr timepoint for NS-LPS vs. NS-SAL in the PVN (*p*_*Bonferroni*_=0.05; d=2.4), PVT (*p*_*Bonferroni*_=2×10^−3^; *d*=3.0), and BLA (*p*_*Bonferroni*_=0.05; *d*=2.0) and for NS-LPS vs. SD-SAL in the PVT (*p*_*Bonferroni*_=0.04; *d*=1.8); and at the 24hr timepoint for NS-LPS vs. SD-SAL in the PVN (*p*_*Bonferroni*_=0.04; *d*=2.0). cFos expression was also reduced at the 24hr timepoint for NS-LPS vs. NS-SAL in the PVT (*p*_*Bonferroni*_=0.02; *d*=2.3). No significant changes in cFos expression were observed at the 0hr timepoint.

For the 6hr timepoint, additional analyses were conducted incorporating the SD-LPS experimental group to examine the relative effects of stressor (NS, SD) and injection type (SAL, LPS). As anticipated, i.p. injections of LPS induced sustained upregulation of cFos expression within all regions (PVN, PVT, BLA) for both NS and SD mice compared to the corresponding SAL controls. Two-way ANOVAs revealed a highly significant effect of LPS vs. SAL injection in the PVN (*F*_*1,20*_=57.7; *p*_*Bonferroni*_<10^−5^), PVT (*F*_*1,23*_=47.4; *p*_*Bonferroni*_<10^−5^), and BLA (*F*_*1,23*_=37.4; *p*_*Bonferroni*_<10^−4^). No significant effect of NS vs. SD stressor was observed in any region (all *p*_*Bonferroni*_>0.1). *Post-hoc* tests (Figure 2B) revealed that both groups receiving LPS, independently of SD exposure, had an increased number of cFos+ neurons per region compared to the corresponding SAL control group: NS-LPS vs. NS-SAL in the PVN (*p*_*Bonferroni*_=0.05; *d*=2.4), PVT (*p*_*Bonferroni*_=2×10^−3^; *d*=3.0), and BAL (*p*_*Bonferroni*_=0.05; *d*=2.0); and SD-LPS vs. SD-SAL in the PVN (*p*_*Bonferroni*_=0.02; *d*=4.3), PVT (*p*_*Bonferron*i_=2×10^−3^; *d*=2.7), and BAL (*p*_*Bonferroni*_=3×10^−3^; *d*=3.4). No differences were detected between the SD-SAL and NS-SAL groups or SD-LPS and NS-LPS groups for any region (all *p*_*Bonferroni*_>0.1).

**Figure 3:**
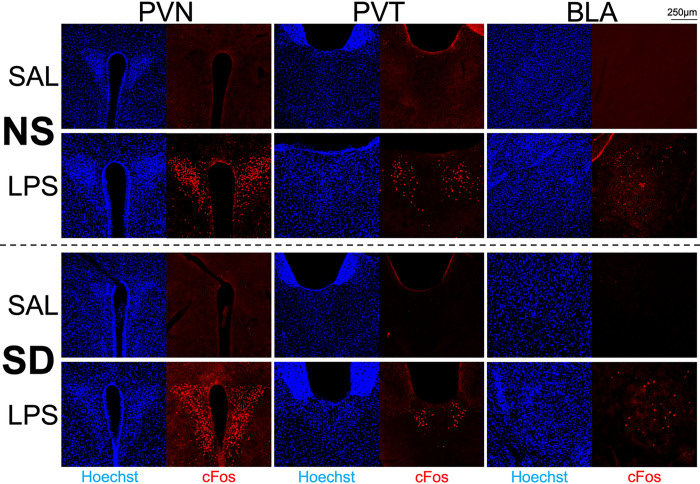
Micrographs showing distribution of all nuclei (Hoechst stain, blue) and cFos+ neurons (red) at 10× magnification. Rows show responses in non-stressed (NS) and reminder social defeat (SD) mice 6hr following i.p. injection with saline (SAL) or lipopolysaccharide (LPS). Columns show the Paraventricular Hypothalamic Nucleus (PVN), Paraventricular Thalamic Nucleus (PVT), and Basolateral Amygdala (BLA) regions of interest.

Representative micrographs for each group and region at the 6hr timepoint are presented in Figure 3. Taken together, our immunohistochemical results indicate that exposure to acute psychosocial stress did not mimic or enhance the sustained effects of acute inflammatory stress on neural activity in juvenile mice.

### Behavioral Validity of Reminder Social Defeat Paradigm

Results from our validity assessment are shown in Figure 2C. During the habituation assay, neither group (reminder SD or no reminder) exhibited a significant preference for either side of the chamber. In the subsequent socialization assay, mice in the no reminder group demonstrated a strong preference for the side with social interaction compared to reminder SD mice (67.7% vs 17.8%; *p*_*Bonferroni*_<10^−4^; *d*=6.7), whereas mice in the reminder SD group preferred the side with the inanimate mouse toy compared to the no reminder group (28.8% vs 17.3% of time; *p*_*Bonferroni*_=2×10^−4^; *d*=2.6).

## DISCUSSION

To our knowledge, this is the first study to investigate immunological responses to the TSST in adolescents. Consistent with our hypothesis, we did not detect increased immune biomarker levels in response to the TSST in our cohort of medication-free adolescents with mood and anxiety symptoms and healthy controls. However, as expected, we were able to document increased levels of multiple inflammatory mediators in response to *in vitro* LPS exposure regardless of TSST exposure, indicating that acute stress had no impact on immune cell competency in this population. These findings were strongly supported by our parallel experiments in juvenile mice exposed to acute social stress and LPS, which yielded highly consistent results and provided insight into the neural mechanisms underlying stress response.

Our findings contrast with the majority of studies employing the TSST in adult populations, which report increased levels of multiple cytokines using a variety of measures, including plasma IL-6 [24, 26–28, 33] [26, 28–30, 35], oral mucosal transudate IL-6 [20], serum IL-6 [23], salivary IL-6 [25], plasma TNF-α [28, 29], plasma IL-1β [28, 29], plasma IL-1RA [21], and plasma IL-10 [29]. The absence of such an inflammatory response to the TSST in adolescents may be related to the lifespan dynamics of the immune system, which entails robust HPA axis activity throughout life but systemic inflammatory responses that build with age [59]. The TSST, as an acute psychological stressor [17], has been consistently documented to increase cortisol secretion [42, 60]. Since cortisol has pronounced anti-inflammatory effects, stress-induced stimulation of the HPA axis may have a proportionally stronger moderating effect on stress-induced immune responses in adolescents. Indeed, prior studies have documented reduced peripheral cytokine responses to the TSST [24] and other acute stressors [61] in individuals with stronger cortisol responses. As clinical studies have shown that cortisol levels rise within 10min of the TSST [17, 32, 62], corresponding to the interval when post-TSST blood samples were collected in our study, cortisol-mediated suppression of an acute inflammatory response is biologically plausible. Notably, we recently found that levels of C-reactive protein (CRP), a biomarker for generalized inflammation, were not associated with psychiatric symptomatology or diagnostic status in a transdiagnostic cohort of 127 adolescents, in contrast to consistent reports of elevated CRP in depressed adults [63]. Taken together, these findings highlight the unique psychosocial and immunological character of adolescence as well as the importance of independently validating biological phenomena observed in adults within this age group.

To help elucidate the neurobiological mechanisms underlying our findings in adolescents, we also conducted a reverse translation experiment using juvenile mice. This parallel approach enabled us to leverage the combined strengths of preclinical techniques (e.g. direct quantification of neural activity using invasive techniques) with those of clinical studies (e.g. greater applicability to target populations). To align the human and animal protocols, we used an acute version of the reminder SD paradigm consisting of a brief sensory re-exposure of socially defeated mice to aggressor mice (Fig. 1A) [49]. Unlike direct SD, which entails components of both physical and social stress, the reminder SD paradigm is devoid of acute physical stress. As such, the reminder SD paradigm in mice provided a reasonable match for the TSST paradigm in humans, generating stress of similar intensity, duration, and social origin.

The response of different brain regions to psychological stress and systemic inflammation was evaluated by examining cFos, a well-established marker of neuronal activity. The cFos response shows clear nuclear signals and thus allows for quantifying the number of activated neurons per brain area under a given experimental condition. Neuronal cFos is induced by both environmental and inflammatory signals. However, in response to stress, cFos induction typically subsides within 1.5hr [64, 65], whereas in response to immune challenge with LPS, cFos induction remains prominent for at least 6hr [66], thus allowing for isolation of inflammation-specific responses from other sources of activity. Consistent with our results in adolescents, the cFos findings in juvenile mice showed a sustained increase in neuronal activity in response to LPS but a lack of sustained effects from SD (Fig. 2A–B), implying that acute psychological stress engages similar circuitry as systemic inflammation but much more transiently. Importantly, our behavioral validity experiment showed that mice that experienced a reminder following initial SD exhibited decreased sociability six hours later compared to mice that received initial SD but no re-exposure (Fig. 2C), demonstrating that the stress paradigm was behaviorally effective. This effect was observed despite the lack of sustained cFos activity in key neuronal circuits responsive to inflammation.

Several limitations should be noted for our clinical study. We collected blood at only a single timepoint 5–10 minutes after the TSST. However, recent work indicates that the temporal dynamics of immune biomarkers vary considerably following acute social stress in adults, with some responses (e.g. IFN-γ, IL-4) peaking within 10 minutes but others peaking later [28]. This concern is partially mitigated by our use of multiplex assays to simultaneously quantify 41 unique biomarkers, which maximized our ability to detect stress-induced immune responses at the selected timepoint. To further characterize potential immune effects at the selected timepoint, we also repeated rANOVA analyses with TSST-induced anxiety levels as an additional covariate, again finding no evidence of an acute stress-related inflammatory response. Future studies should collect additional timepoints to better resolve any delayed immune effects. Another limitation was that we did not monitor NF-κB, cortisol, or sensitivity to glucocorticoids. As HPA axis dysregulation is linked with psychopathology in adolescents [42, 59], a key area of future research will be to characterize the interplay between hormonal and immunological responses to acute stress in youth. The insertion of the cannula for blood collection may also have served as a stressor on participants, independent of the TSST; future work should include measures of cortisol as well as affective response pre- and post-insertion to account for such effects. Finally, though well-powered to detect moderate or larger effects, our adolescent sample was modest and included a subset of healthy controls, which may have influenced results. Although it is conceivable that results would have been more pronounced in an exclusively psychiatric cohort, our decision to include control subjects was driven by our adherence to an RDoC approach, which posits that psychiatric conditions exist on a spectrum of severity that extends into nominally healthy populations. Considering our negative results in this mixed adolescent cohort, more work is needed to characterize immune activation and its association with specific clinical conditions in youth.

Regarding our animal experiments, it should be noted that we focused only on neuronal responses to social stress and peripheral inflammation in our main experiment. These stressors may also have induced behavioral responses of potential interest, as seen in our behavioral validity experiment. SD and LPS induced cFos activation in similar brain areas; however, sustained cFos activity was only observed in the LPS but not SD group, suggesting that the effects of SD did not involve the induction of systemic inflammation. Thus, it is unlikely that SD-induced disruption of sociability was mediated by peripheral inflammation. Another potential concern relates to statistical power, with sensitivity analyses indicating limited ability to detect moderate or smaller group and timepoint effects in our animal study. Despite this, the effects of LPS on cFos expression were readily apparent, highlighting the robust neuronal response to acute immune challenge in the targeted brain regions. As such, any neuronal response to acute social stress that may occur in juvenile mice must be far less pronounced – exactly the pattern we observed for peripheral inflammatory responses in the adolescent clinical study.

In conclusion, we conducted a detailed investigation of immune and neuronal responses to acute social and inflammatory challenges in youth across species. Our focus on immune response in adolescents with diverse psychiatric symptomatology was motivated by the dual facts that psychiatric conditions often begin early in life and entail chronic inflammation. If a differential immune response can be evoked in adolescents with clinical symptoms, those most at risk based on their immune phenotype may be identified for earlier and more targeted interventions. Our results indicate that the TSST is not a promising candidate for evoking such immune responses in adolescents. In a parallel experiment, we examined markers of neuronal activity within key stress-related brain regions in juvenile mice following the reminder SD paradigm, an acute social stressor. Findings were highly consistent with our adolescent TSST results, indicating no sustained impact of stress on neuronal activity. Importantly, both our *in vitro* human and *in vivo* animal experiments included an immune challenge with LPS, which confirmed our ability to detect acute inflammatory responses using these protocols. As expected, adolescent blood samples cultured with LPS exhibited robust induction of proinflammatory cytokines, while mice injected with LPS showed a much higher level of neuronal activity within the selected brain regions. Since our negative TSST findings in adolescents are at odds with reports of TSST-induced immune activation in adults, we speculate that aging, chronic stress, and chronic inflammation may account for the stronger effects of social stress on immune processes in adults. Future work will explore the interplay between HPA- and cytokine-mediated immune responses as well as the temporal dynamics of adolescent stress responses.

## Figures and Tables

**Table 1:** Participant Characteristics

Total Participants (N)	31
with Psychiatric Symptoms	25 (81%)
Healthy Controls	6 (19%)
Age (years)	15.1 ± 2.2
BMI (kg/m^2^)	24.3 ± 5.3
Gender	
Female	15 (48%)
Male	16 (52%)
Race	
White	14 (45%)
Black	11 (35%)
Asian	1 (3.2%)
Multiracial/Other	5 (16%)
Ethnicity	
Hispanic	12 (39%)
Non-Hispanic	19 (61%)
Depression Severity (CDRS-R)	38.6 ± 19.0
Depression Severity (BDI)	14.5 ± 13.0
Anxiety Severity (MASC)	44.1 ± 20.6
Anxiety during TSST (1–6 VAS)	4.0 ± 1.5

Data reported as n (%) or Mean ± SD.

**Table 2:** Effects of TSST and LPS on Immune Biomolecules in Adolescents

Biomolecule	TSST		LPS		TSST×LPS	
	*p*	*f*	*p*	*f*	*p*	*f*
**EGF**	0.70	5.6×10^−3^	0.45	2.3×10^−2^	0.73	4.5×10^−3^
**Eotaxin**	0.94	2.3×10^−4^	0.33	3.6×10^−2^	0.68	6.5×10^−3^
**FGF-2**	0.43	2.4×10^−2^	**9.5×10^−9^**	**1.1**	0.53	1.5×10^−2^
**Flt-3L**	0.45	2.2×10^−2^	**1.3×10^−10^**	**1.3**	0.93	3.3×10^−4^
**Fractalkine**	0.056	0.13	**6.2×10^−8^**	**0.93**	0.99	1.3×10^−5^
**G-CSF**	0.60	1.1×10^−2^	0.39	2.8×10^−2^	0.28	4.6×10^−2^
**GM-CSF**	0.43	2.4×10^−2^	**1.4×10^−10^**	**1.3**	0.85	1.5×10^−3^
**GRO**	0.78	3.1×10^−3^	0.56	1.3×10^−2^	0.95	1.4×10^−4^
**IFNa2**	0.72	5.2×10^−3^	**7.6×10^−11^**	**1.4**	0.92	3.9×10^−4^
**IFNg**	0.68	6.8×10^−3^	**2.5×10^−7^**	**0.85**	0.78	3.2×10^−3^
**IL-1a**	0.43	2.4×10^−2^	**1.9×10^−9^**	**1.2**	0.37	3.2×10^−2^
**IL-1b**	0.83	1.8×10^−3^	**3.5×10^−4^**	**0.43**	0.81	2.2×10^−3^
**IL-1RA**	0.61	1.0×10^−2^	**2.7×10^−4^**	**0.44**	0.80	2.5×10^−3^
**IL-2**	0.70	5.9×10^−3^	**7.0×10^−97^**	**>5.0**	0.83	1.7×10^−3^
**IL-3**	0.60	1.1×10^−2^	**9.1×10^−7^**	**0.77**	0.83	1.7×10^−3^
**IL-4**	0.60	1.1×10^−2^	**3.1×10^−14^**	**2.0**	0.43	2.4×10^−2^
**IL-5**	0.35	3.4×10^−2^	**7.0×10^−4^**	**0.39**	0.35	3.4×10^−2^
**IL-6**	0.56	1.3×10^−2^	**1.1×10^−3^**	**0.36**	0.54	1.4×10^−2^
**IL-7**	0.23	5.5×10^−2^	0.15	7.7×10^−2^	0.80	2.6×10^−3^
**IL-8**	0.89	8.1×10^−4^	**3.2×10^−4^**	**0.43**	0.40	2.7×10^−2^
**IL-9**	0.68	6.7×10^−3^	**1.7×10^−16^**	**2.5**	0.63	9.1×10^−3^
**IL-10**	0.86	1.1×10^−3^	**2.6×10^−9^**	**1.1**	0.98	3.0×10^−5^
**IL-12P40**	0.62	9.5×10^−3^	**6.9×10^−11^**	**1.4**	0.99	2.2×10^−6^
**IL-12P70**	0.81	2.2×10^−3^	**6.8×10^−10^**	**1.2**	0.87	1.0×10^−3^
**IL-13**	0.61	1.0×10^−2^	0.033	0.17	0.30	4.1×10^−2^
**IL-15**	0.52	1.6×10^−2^	**6.1×10^−17^**	**2.6**	0.40	2.7×10^−2^
**IL-17A**	0.55	1.4×10^−2^	**3.8×10^−4^**	**0.42**	0.83	1.8×10^−3^
**IP-10**	0.31	4.0×10^−2^	0.020	0.19	0.26	4.9×10^−2^
**MCP-1**	0.20	6.2×10^−2^	0.59	1.1×10^−2^	0.74	4.4×10^−3^
**MCP-3**	0.15	7.7×10^−2^	0.15	8.0×10^−2^	0.34	3.5×10^−2^
**MDC**	0.54	1.5×10^−2^	0.015	0.21	0.62	9.7×10^−3^
**MIP-1a**	0.61	1.0×10^−2^	0.10	0.10	0.57	1.3×10^−2^
**MIP-1b**	0.28	4.4×10^−2^	0.083	0.11	0.28	4.6×10^−2^
**PDGF-AA**	0.86	1.2×10^−3^	0.49	1.8×10^−2^	0.58	1.2×10^−2^
**PDGF-AB/BB**	0.69	6.1×10^−3^	0.33	3.7×10^−2^	0.63	8.8×10^−3^
**RANTES**	0.32	3.8×10^−2^	0.39	2.9×10^−2^	0.33	3.6×10^−2^
**sCD40L**	0.99	1.0×10^−5^	0.72	5.1×10^−3^	0.76	3.7×10^−3^
**TGF-a**	0.72	5.1×10^−3^	**2.4×10^−6^**	**0.71**	0.98	3.0×10^−5^
**TNFa**	0.99	8.3×10^−6^	0.018	0.20	0.98	2.9×10^−5^
**TNFb**	0.81	2.3×10^−3^	0.58	1.2×10^−2^	0.83	1.9×10^−3^
**VEGF**	0.48	1.9×10^−2^	0.020	0.20	0.26	4.9×10^−2^

Significance (two-tailed *p*) and effect size (Cohen’s *f*) for effects of TSST, LPS, and TSST×LPS interaction in 2×2 rANOVA, controlled for age, sex, BMI, and diagnostic status. Effect sizes converted from *η*_*p*_^2^ to *f* for ease of interpretation. Findings that passed Bonferroni correction (*p*<0.05/41≈1.2×10^−3^) indicated in **bold**.
